# A Protocol for Extraction of Infective Viromes Suitable for Metagenomics Sequencing from Low Volume Fecal Samples

**DOI:** 10.3390/v11070667

**Published:** 2019-07-20

**Authors:** Ling Deng, Ronalds Silins, Josué L. Castro-Mejía, Witold Kot, Leon Jessen, Jonathan Thorsen, Shiraz Shah, Jakob Stokholm, Hans Bisgaard, Sylvain Moineau, Dennis Sandris Nielsen

**Affiliations:** 1 Section of Food Microbiology and Fermentation, Department of Food Science, Faculty of Science, University of Copenhagen, Rolighedsvej 26, 1958 Rederiksberg, Denmark; 2 Department of Environmental Science, Aarhus University, Frederiksborgvej 399, 4000 Roskilde, Denmark; 3 Copenhagen Prospective Studies on Asthma in Childhood (COPSAC), Herlev and Gentofte Hospital, University of Copenhagen, Ledreborg Alle 34, 2820 Gentofte, Denmark; 4 Département de biochimie, de microbiologie, et de bio-informatique, Faculté des sciences et de génie, Université Laval, Québec City, QC G1V 0A6, Canada; 5 Félix d’Hérelle Reference Center for Bacterial Viruses and Groupe de recherche en écologie buccale, Faculté de médecine dentaire, Université Laval, Québec City, QC G1V 0A6, Canada

**Keywords:** human gut phageome, human gut virome, microbiome, isolation, purification, phage, T4, c2, phiX174, phi29

## Abstract

The human gut microbiome (GM) plays an important role in human health and diseases. However, while substantial progress has been made in understanding the role of bacterial inhabitants of the gut, much less is known regarding the viral component of the GM. Bacteriophages (phages) are viruses attacking specific host bacteria and likely play important roles in shaping the GM. Although metagenomic approaches have led to the discoveries of many new viruses, they remain largely uncultured as their hosts have not been identified, which hampers our understanding of their biological roles. Existing protocols for isolation of viromes generally require relatively high input volumes and are generally more focused on extracting nucleic acids of good quality and purity for down-stream analysis, and less on purifying viruses with infective capacity. In this study, we report the development of an efficient protocol requiring low sample input yielding purified viromes containing phages that are still infective, which also are of sufficient purity for genome sequencing. We validated the method through spiking known phages followed by plaque assays, qPCR, and metagenomic sequencing. The protocol should facilitate the process of culturing novel viruses from the gut as well as large scale studies on gut viromes.

## 1. Introduction

During the past decades it has become apparent that the human gut microbiome (GM) has profound influence on the states of health and disease. While most studies investigating the human GM have focused on the bacterial component, there is an emerging understanding that non-bacterial members (archaea, eukaryotes, and viruses) have deep impacts on GM structure and function [1,2,3], as well as host health [4,5,6,7], especially with the viruses playing a significant role. 

Advances in metagenomics have led to a rapid and massive expansion in the known diversity of viral genomes, but most of these have no identified host, and the knowledge of their characteristics is very limited [8,9,10]. While metagenomics is indispensable for the discovery of new viral genomes, functional virology research requires isolation of cultivable viruses and their hosts. Development of efficient protocols for purification of infective viromes from fecal samples is thus essential for detailed studies coupling bacterial hosts and phages. Moreover, many of the reported methods for fecal virome extraction require gram-scale input and long processing times [11,12,13,14]. Importantly, these protocols are usually constrained by the number of samples that can be processed in parallel, which makes large scale studies very tedious. 

With the aim of enabling isolation and characterization of infective gut viromes for large scale studies and studies where limited input material is available (i.e., limited biobanked fecal samples or rodent fecal samples), we report the development of an efficient protocol for the extraction of infective viruses from low volume fecal samples. The isolation of infective phages was validated by spiking the fecal samples with known phages from different viral families and determining phage recovery rates during purification by plaque assays and qPCR. Finally, the extracted viromes were analyzed by shot gun sequencing. 

## 2. Materials and Methods 

### 2.1. Sample Collection and Storage

Fecal samples were obtained from three anonymous healthy human infants aged ~1 year. The samples were collected at the infants’ homes, mixed equally with 2× SM buffer (400 mM NaCl, 20 mM MgSO_4_, 100 mM Tris-HCl, pH 7.5) containing 30% glycerol in 50 mL tubes and preserved in cooler bags with ice-packs (temperature 2–5 °C). Samples were delivered to the laboratory within 16 h, upon reception immediately divided into smaller aliquots (0.5 g) and stored at −80 °C until further use.

### 2.2. Virus Stock Production

The protocol was optimized and validated by spiking fecal samples with known viruses representing four common phage families namely *Podoviridae* (phage Φ29), *Myoviridae* (phage T4), *Siphoviridae* (phage c2), and *Microviridae* (phage ΦX174) (Table 1). *Lactococcus lactis* MG1363, the host of phage c2, was grown in M17 broth (Merck, Kenilworth, NJ, USA) containing 5 mM CaCl_2_ and 0.4% glycine at 30 °C. Phage Φ29’s host, *Bacillus subtilis* DSM 5547, was grown in TS broth (Merck, Kenilworth, NJ, USA) at 37 °C while shaken at 225 rpm. The host of phage T4, *Escherichia coli* DSM 613, was grown in LB broth (Merck, Kenilworth, NJ, USA) at 37 °C while shaken at 225 rpm. *E. coli* ATCC 13706, the host of phage ΦX174, was grown in BHI broth (Merck, Kenilworth, NJ, USA) at 37 °C while shaken at 225 rpm. 

For virus propagation, 100 µL of bacterial overnight culture was added to 2× 10 mL of broth (Table 1), and grown for 2 h at 37 °C with shaking at 225 rpm, except for *Lactococcus lactis* MG 1363 which was grown at 30 °C without shaking. After incubation, 50 µL of the respective phage stock lysate was added to one tube of each pair and both tubes were further incubated overnight. The following day, the lysed cultures were transferred to a 50 mL tube and centrifuged at 5000× *g* for 30 min at 4 °C to remove cell debris. The supernatant was recovered and filtered through a 0.45 µm syringe filter and stored at 4 °C. Infective phages in the filtrate were enumerated by plaque assay. 

### 2.3. Spiking of Fecal Samples with Known Phages

Fecal samples were diluted with 30 mL SM buffer (in a 50 mL centrifuge tube, Sarstedt, Nümbrecht, Germany) and spiked with each phage (Table 1) to a final concentration of 10^4^ plaque forming units per milliliter (PFU/mL) respectively. Phage lysates were diluted with SM buffer to obtain the desired titer prior to spiking. 

### 2.4. Plaque Assay

To quantify the recovered phages at different purification steps, plaque assays were performed [11]. Prior to plaque assays, spots assays with dilution to plaques were applied to determine the optimal dilution level for plating. Briefly, 5 mL of media containing 0.5% agarose pre-warmed at 37 °C was mixed with 100 µL of the diluted phage sample and 200 µL of the bacterial culture and poured to the top of a pre-warmed agar plate (1.5%). The double layer plates were first solidified at room temperature and then incubated overnight at the corresponding growth temperature of the bacterial host. On the next day the phage plaques were counted and PFU/mL calculated. 

### 2.5. Virome Isolation from Feces

After spiking with known phages, samples were poured into a stomacher filter bag (Interscience BagPage, 100 mL, Saint-Nom-la-Bretèche, France). The mixture was homogenized (Stomacher 80, Seward, UK) for 120 s at the high level setting. Homogenized samples, from the other side of the filter in the bag, were transferred to 50 mL tubes and centrifuged at 5000× *g* for 30 min at 4 °C. After centrifugation, the supernatant was filtered through a 0.45 µm PES filter (Minisart^®^ High Flow Syringe Filter, Sartorius, Göttingen, Germany) into the bottom of the outer tube of a Centriprep 50K device ( Millipore, Burlington, MA, USA). Afterwards, the filtrate was purified and concentrated using the Centriprep 50K device by centrifuging at 1500× *g* three times in a row, first time for 30 min, second time for 10 min, and third time for 3 min. Extra centrifugation time was sometimes applied to allow the liquid level in the inner tube to be similar to the outer tube. The liquid filtered into the inner tube was poured off after each centrifugation step. A volume of 200 µL SM buffer was added to the inner tube at the end and centrifuged for 3 min. After the final centrifugation, 140 µL of the concentrated virome solution remaining in the outer tube was collected. The Centriprep filter membrane was cut out and added to the virome solution before storing at −80 °C until nucleic acids extraction. The remaining volume was stored at 4 °C for plaque assays. 

### 2.6. Nucleic Acid Extraction of Virome from Feces

The concentrated virome solution and the cut filter membrane was first treated with 1 µL of 100 time diluted Pierce™ Universal Nuclease (Thermofisher Scientific, Waltham, MA, USA) for 5 min at room temperature, then the QIAmp viral RNA mini kit (Qiagen, Hilden, Germany) was used for viral DNA/RNA extraction following the procedures described by the manufacturer with modifications as described in [15]. Next, 10 µL of the extracted nucleic acids were amplified through Multiple Displacement Amplification (MDA) using the Genomephi V3 kit (GE Healthcare Life Sciences, Marlborough, MA, USA) following the instructions of the manufacturer, but the amplification time was shortened to 30 min (from 90 min). Finally, the amplified DNA was cleaned using a Genomic DNA Clean & Concentrator™ Kit (Zymo Research, Irvine, CA, USA) following the manufacture’s protocol.

### 2.7. Virus Quantification by Quantitative Real-Time PCR (qPCR)

Phage T4 was also quantified by real-time qPCR using SYBR Green Master Mix (Roche, Basel, Switzerland) on 7500 Fast Real-Time PCR System (Applied Biosystems, Foster City, CA, USA). Five pmol of forward and reverse primers (5′-CACAGAGGAACGGTCTTGTAAA-3′ and 5′-GAGAAGCCCTCCAGAATCATAAA-3′ targeting the T4 genome from position 53,921 to 54,070 amplifying a 150 bp fragment) were added to 20 µL reactions, which were run using the following setup: initial stage at 50 °C for 2 min, hot start at 95 °C for 2 min, followed by 40 cycles of (i) 95 °C for 15 s, (ii) 55 °C for 30 s, and (iii) 72 °C for 30 s [16]. Serial five-times dilutions of T4 genomic DNA were used to generate standard curves. After the qPCR amplification, a melting curve analysis was performed in order to distinguish putative nonspecific amplifications. Each reaction was performed in duplicates.

### 2.8. Sequencing of Fecal Virome Nucleic Acids

The concentration of the MDA amplified and cleaned DNA was measured by Qubit dsDNA HS Assay Kit (ThermoFisher Scientific, Waltham, MA, USA). Random shotgun libraries were constructed using the Nextera XT kit (Illumina, San Diego, CA, USA) and normalized by AMPure XP beads following the standard procedures described by the manufactures. Constructed libraries were sequenced using 2  ×  150 bp paired-end settings on an Illumina NextSeq platform.

### 2.9. Processing and Analysis of the Sequencing Results

The sequencing data obtained were processed and analyzed using a pipeline previously described in [17]. Briefly, the raw reads were trimmed using Trimmomatic v0.35 (>97%). As quality control, presence of non-viral DNA was quantified using 50,000 random forward-reads from each sample, which were queried against the human genome, as well as all the bacterial and viral genomes hosted at NCBI using Kraken2 [18]. For each sample, reads generated from virus-like particles (VLPs)-derived DNA sequencing were subjected to within-sample de novo assembly using Spades v3.5.0 [19] and contigs with a minimum length of 1000 nt were retained. Contigs generated from all samples were pooled and de-replicated by multiple blasting and removing those contained in over 90% of the length of another (90% similarity), as outlined previously [20]. Following assembly and quality control, high-quality/de-replicated reads from all samples were merged and recruited against all the assembled contigs at 95% similarity using Subread [21] and a contingency-table of reads per Kbp of contig sequence per million reads sample (RPKM) was generated. Taxonomy assignment was performed using the Contig Annotation Tool (CAT) [22] applying 0.1 from the highest bitscore for LCA (last common ancestor) identification of individual ORFs, 0.5 for the maximum achievable bitscore for the contig and a minimum alignment quality (bitscore value) of 200. The reference database was RefSeq Virus as for November 2018.

## 3. Results and Discussion

### 3.1. Design of the Experiments

**Figure 1:**
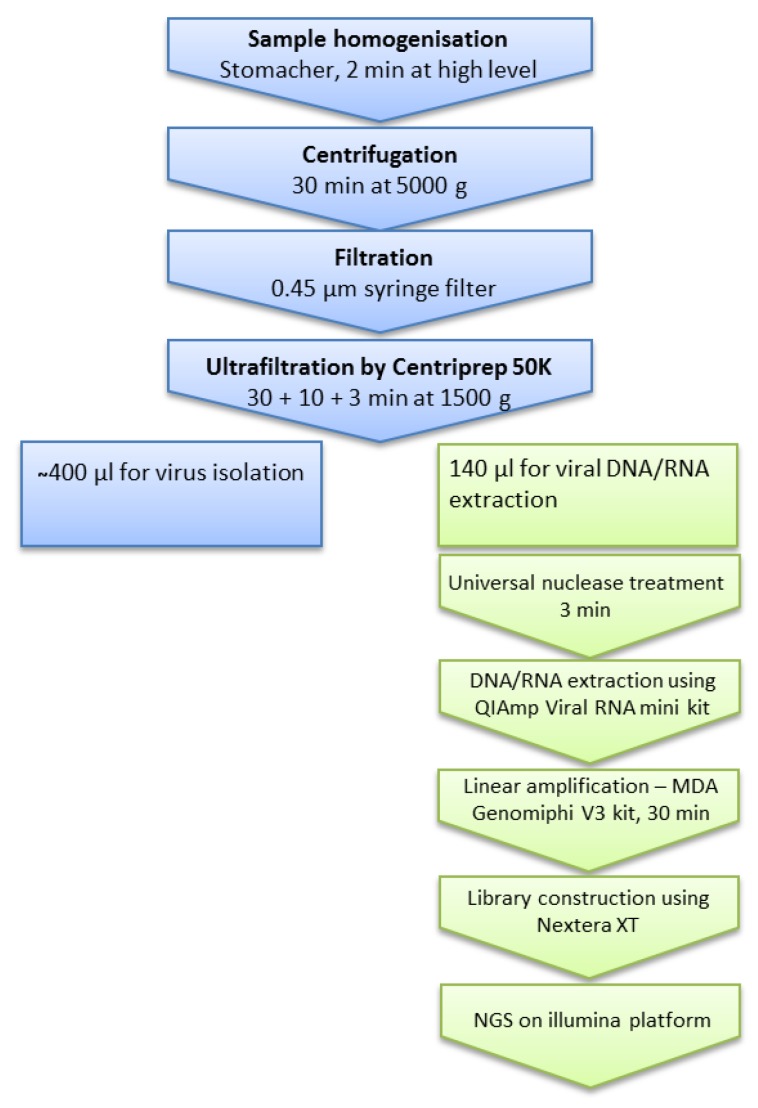
Overview of the virome extraction, amplification, and sequencing procedures. A workflow for gut virome extraction and sequencing was established, the virome isolation part and sequencing part is in blue and green, respectively.

Since we aimed to isolate infective phages simultaneously with nucleic acids suitable for downstream processing, caution was taken when designing a process where not only the phage particles should be kept intact, but also the receptor-binding fibers used to bind to their bacterial hosts. Taking advantage of the possibility to concentrate VLPs using Centriprep-filters, we chose an approach where the low-input fecal samples (containing approximately 250 mg fecal matter) were first diluted with 30 mL of buffer before the first homogenization step (Figure 1).

Figure 1 shows that bacterial cells and other larger particles were pelleted by centrifugation at a modest speed (5000× *g*), and the supernatant subsequently cleaned by gentle filtration through 0.45 μm pore polyethersulfone (PES) membrane filters. We chose to use 0.45 µm PES filters for easier filtration and maximal recovery of the phages while ensuring removal of bacterial cells [15]. Then, viral particles from the filtrate were concentrated by ultrafiltration using Centriprep 50K tubes to a final volume of approximately 550 µL. Depending on the centrifuge, 16 viromes can be isolated and concentrated simultaneously on a Beckman Allegra 25R refrigerated centrifuge and 24 on an Eppendorf 5920 centrifuge. The total processing time in both cases was less than 4 h, with a hands-on time less than 2 h making extraction of 48 samples feasible in one work day with less than 4 h of hands-on time. 

Cesium chloride (CsCl) density gradient centrifugation can yield VLPs of high purity, but was avoided here as it is known to damage phages with fragile tail structures [11,23]. Moreover, CsCl density gradient centrifugation is labor intensive and requires lengthy centrifugation steps, and consequently the number of samples that can be processed simultaneously is limited [11,12,14]. PEG/NaCl precipitation was also not selected here to concentrate viral particles as optimal PEG/NaCl concentration for precipitation is phage-dependent [24], and using this method could introduce bias into the viral populations after recovery. It has also been reported that chloroform can be added to disrupt the cell membrane, allowing further removal of bacteria and its debris, but enveloped viruses would be removed at the same time [12,13,14,20,23]. Therefore, we chose not to treat the virome samples with chloroform.

### 3.2. Assessing the Protocol Design by Recovery Rates of Spiked Phages

**Figure 2:**
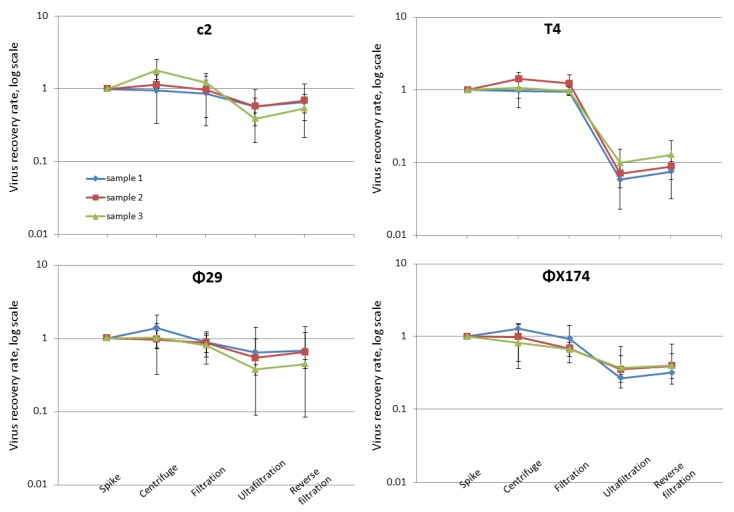
Infective phage recovery determined by plaque assays. The percentages of phages recovered (y axis) were determined by plaque assay at each different sampling point (x axis). The error bars here indicate the standard deviation of 3 replicates.

Most published virome extraction protocols from fecal samples do not consider the loss of infectivity during the purification procedures [25,26,27,28], and those that do, require rather high volumes of the fecal sample [11]. To estimate the loss of phage infectivity during our proposed extraction procedure, phages T4 (Myoviridae), c2 (Siphoviridae), Φ29 (Podoviridae), and ΦX174 (Microviridae) representing the four most abundant phage families in the human gut were spiked into fecal samples and their recovery rates at the different steps of the extraction protocol were determined by plaque-assays [29]. We first confirmed that no plaques were formed with the host strains (Table 1) using the viromes prepared from non-spiked fecal samples (results not shown). The average final recovery rates for infective phages were on average 63.1% (±6.4%), 9.8% (±2.2), 59.1% (±10.4%), and 29.4% (±9.2%) for c2, T4, Φ29, and ΦX174, respectively, with the majority loss of infectivity happening during the ultrafiltration procedure (Figure 2). However, a helpful feature of the Centriprep ultrafilter is that it allows reverse flow of the buffer through the membrane when the liquid level of the inner tube is higher than that of outer tube, which can wash the attached viruses off the filter membrane. We observed a 2–10% increase in the final phage recovery rate after this step. The highest loss (1 log) of infective particles was observed with phage T4. The loss of phage T4 infectivity during extraction from fecal samples is a common challenge and may reflect damage of the fragile fiber structure as suggested earlier [11]. Importantly, the recovery of approximately 10% of infective phages for T4 phages here was at least an order of magnitude higher than in previously published protocols [11].

### 3.3. Determination of T4 Genome Recovery Rate by qPCR

The reduction of infective T4 numbers may mainly be due to the damage of its fragile structure, but it could also because the entire viral particles were lost during the purification process. Therefore, T4-specific qPCR was performed to determine the recovery rate of T4 genomes, as the genomes should still be present as long as the capsid is intact. In accordance with our previous observation [11], the final recovery rate of T4 genomes is much higher when determined by qPCR. For sample 1, 2, and 3, the recovery rate was 21.6% (±1.4%), 72.2% (±4.8%), and 65.4% (±2.6%), respectively. The large increase for all the samples suggested that T4 phages mainly lost infectivity during the purification, but their capsids were kept intact as the genome can still be detected [11].

### 3.4. Assessing the Protocol by Sequencing and Bioinfomatic Analysis

**Figure 3:**
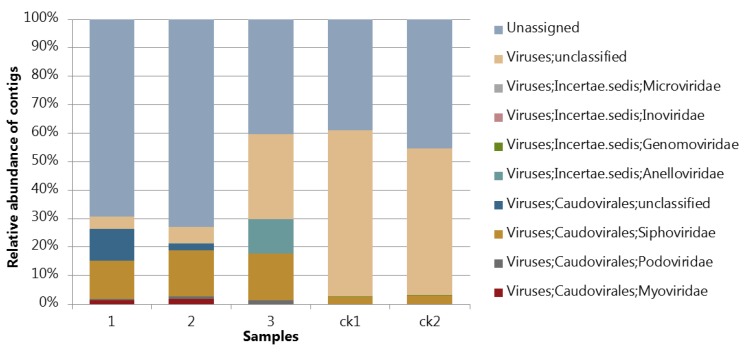
Taxonomic distribution (relative abundance) of the sequenced viromes. The relative distribution is described at the taxonomical level of orders. Taxonomy of contigs was determined by querying the viral contigs against a database containing taxon signature genes for virus orthologous group hosted at www.vogdb.org. The unassigned category is the contigs that have no relation to any known classified sequences.

**Figure 4:**
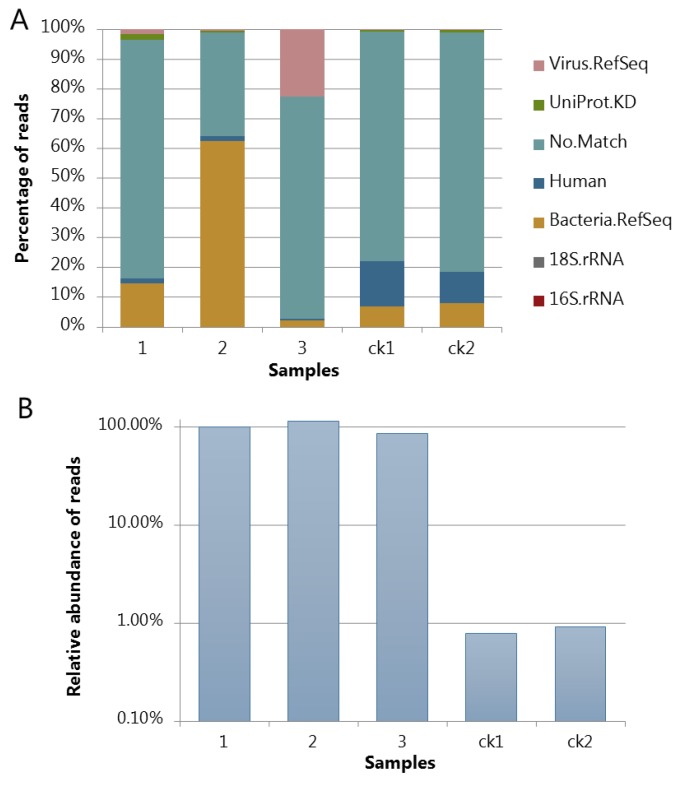
(**A**) Distribution of sequencing reads into the different taxonomic categories viral, human, bacterial, and unknown origin. To check the presence of non-viral DNA sequences, 50,000 random forward reads were evaluated according to their match to a range of viral, bacterial, and human reference genome and protein databases as described in [17]. No reads (in 50,000 reads) matched to the 16S rRNA gene sequences in all the samples. (**B**) Relative abundance of sequencing reads matching the assembled virus-like contigs compared to the average of the three true samples. At least 10 times coverage/contig was applied here as the threshold for counting. Numbers 1–3refer to viromes extracted from feces from infants 1–3. ck1 and ck2 refer to co-extracted blank (SM buffer) samples.

After the VLPs were concentrated from the fecal samples, viral DNA was extracted and amplified by MDA to include single strand DNA (ssDNA) viruses during library construction and sequencing. Only a half hour incubation was performed instead of 1.5 h as described in the standard protocol for MDA to limit the selective amplification of ssDNA, which is known to increase with incubation time [27]. As seen from Figure 4, only a minor fraction of the metavirome sequences was derived from human, fungi, or bacterial genomes, indicating that the method is selective in separating viral particles and larger particles such as bacteria. No 16S rRNA gene fragments were detected in 50,000 reads in any of the samples underlining that the protocol is efficient in removing bacterial cells and genomic fragments. However, as seen from Figure 4A, the fecal sample from infant 2 was found to contain a rather high fraction of reads aligning to bacterial genomes, but a closer analysis of the results showed that many of these reads matched to putative prophage sequences in *Bacteroides dorei*. The *B. dorei* cell size has been reported to be 1.6–4.2 µm by 0.8–1.2 µm [30], meaning that it should not pass through the 0.45 µm filter. Moreover, the samples from infants 1 and 3 showed very few hits aligning to the bacterial genomes, reflecting that the there was no systematic bacterial contamination due to the extraction protocol. Moreover, the detected bacterial hits may reflect that the abundance of induced prophages varied among different samples. Negative controls (SM buffer control, ck1 and ck2; Figure 3 and Figure 4) were also sequenced. As seen from Figure 4B, the number of reads matching viral-like sequences were less than 1% of the true samples and with a composition much different from the fecal samples (Figure 3) where Caudovirales was the dominant order as found in most infant gut viromes [20,29].

Several host prediction bioinfomatic softwares could be employed to identify the potential hosts of these viral contigs [31,32,33]. Subsequently, plaque assays against the predicted host(s) can be carried out which will facilitate the pairing of targeted viral particles and host.

In summary we here describe a protocol for extraction of viromes with infective capacity from low volume fecal samples suitable for metagenomic sequencing. The protocol has a relatively high throughput allowing extraction of up to 48 viromes within one working day and with less than 4 h of hands-on time.

## Figures and Tables

**Table 1:** Bacterial strains and their respective bacteriophages.

Bacterial Strain	Phage (Family)	Growth Media	Source
*Bacillus subtilis* DSM 5547	Φ29 (*Podoviridae*)	TSB	Lab.stock
*Escherichia coli* DSM 613	T4 (*Myoviridae*)	LB medium	Lab.stock
*Escherichia coli* ATTC 13706	ΦX174 (*Microviridae*)	BHI Broth	Félix d’Hérelle Reference Center
*Lactococcus lactis* MG1363	c2 (*Siphoviridae*)	M17	Lab. Stock
